# Ephemeroptera, Plecoptera and Trichoptera (Insecta) Abundance, Diversity and Role in Leaf Litter Breakdown in Tropical Headwater River

**DOI:** 10.21315/tlsr2017.28.2.7

**Published:** 2017-07-31

**Authors:** Suhaila Ab Hamid, Che Salmah Md Rawi

**Affiliations:** School of Biological Sciences, Universiti Sains Malaysia, 11800 USM, Pulau Pinang, Malaysia

**Keywords:** Leaf Packs, Tropical River, *Pometia pinnata*, *Dolichandrone spathacea*, Leaf Breakdown

## Abstract

Leaf litter decomposition in a tropical stream was examined in two types of leaf packs; single species leaf packs of *Pometia pinnata* and two species leaf packs of equal combination of *Pometia pinnata* and *Dolichandrone spathacea* leaves. Both leaf packs were immersed in a river and weekly examined for remains of decomposed leaves and presence of EPT. In the control leaf packs, leaves in the two species leaf packs treatments decomposed within 35 days, faster than in single species leaf packs which decomposed after 42 days. In the presence of EPT, the leaf breakdown took 28 days in two species and 35 days for single species leaf packs. Higher abundance of EPT was observed in single species leaf packs but its diversity was higher in two species leaf packs. Litter breakdown in the stream was faster in the presence of EPT and softer leaves of *D. spathacea* with higher nitrogen content underwent faster decomposition and sustained higher numbers of EPT.

## Abstract (ru)

Penguraian longgokan daun di dalam sungai tropika diperiksa dalam dua jenis pek daun; Pek daun spesis tunggal *Pometia pinnata* dan pek daun dua spesis yang sama dengan kombinasi: *Pometia pinnata* dan daun *Dolichandrone spathacea*. Kedua-dua pek daun telah direndam di dalam sungai dan diperiksa setiap minggu untuk sisa-sisa daun terurai dan kehadiran EPT. Dalam pek daun kawalan, daun dalam pek dua spesies rawatan daun terurai dalam masa 35 hari, lebih cepat daripada pek daun spesies tunggal yang terurai selepas 42 hari. Pecahan daun mengambil masa 28 hari dalam dua spesis dan 35 hari untuk pek daun spesies tunggal dengan kehadiran EPT. Kelimpahan EPT yang lebih tinggi diperhatikan dalam pek daun spesis tunggal tetapi kepelbagaiannya lebih tinggi dalam dua pek daun spesis. Penguraian daun di sungai lebih cepat dengan kehadiran EPT dan daun lembut *D.spathacea* kandungan nitrogen yang lebih tinggi akan mengalami penguraian lebih cepat dan dapat mengekalkan jumlah EPT yang lebih tinggi.

## INTRODUCTION

Aquatic insects especially those in the orders Ephemeroptera, Plecoptera and Trichoptera (EPT) play an important role in decomposition of riparian leaf litters in forest streams (Anderson & Sedell 1979; Webster & Benfield 1986; Voshell & Reese 2002; Merritt *et al*. 2008). Likewise, Knopf and Samson (1994), Lachavanne and Juge (1997), and Iwata *et al.* (2003) reported that riparian trees in the forests often support diverse and abundant communities of invertebrates especially aquatic insects in adjacent streams because leaf litters provide a significant source of energy and nutrients for headwaters invertebrates (Graca 2001; Colon-Gaud *et al*. 2008). Some stream shredder insects such as calamoceratid caddisflies use leaf litters as food source as well as habitats (Dudgeon & Wu 1999). Disturbance in riparian forests especially along the riverbanks can greatly influence the timing, quantity and quality of allochthonous coarse particulate organic matter (CPOM) inputs into streams (Sweeney 1993; Tuchman & King 1993). Changes in riparian vegetation considerably influence macroinvertebrate communities because of their strong dependence on allocthonous food base (Cummins & Klug 1979; Hawkins *et al*. 1982).

Leaf shredders detritivores (mainly Plecoptera and Trichoptera) in low order streams feed directly on CPOM and their feeding activity is an important mechanism in conversion of organic particles from CPOM to fine particulate organic matter (FPOM). The FPOM is subsequently used as food by collector-gatherers and collector-filterers (Wallace & Merritt 1980). Although macroinvertebrates quickly colonize fallen leaves (Dudgeon 1982; Stewart 1992), some of them, including EPT have strong preferences over leaf species (Canhoto & Graca 1999; Mathuriau & Chauvet 2002). Consequently, Webster and Benfield (1986) reported that riparian vegetation determined the allocthonous organic matters in the river, which in turn affected the abundance and diversity of resident aquatic insects (Mackay & Kersey 1985).

Although the influences of benthic macroinvertebrates and aquatic hypomycetes abundance and diversity on leaf litter decomposition process are well documented (Whiles & Wallace 1997), the effects of EPT abundance and diversity on the process are less understood. In this study, the importance of EPT in forest leaf litter breakdown was investigated by comparing the litter breakdown rate in control leaf packs (in the absence of EPT) and in experimental leaf packs with free access to EPT. It is known that most macroinvertebrates are shredders, however litter breakdown in upland rivers might be helped by the EPT as these three orders was known dominating the upland rivers in diversity and abundance. The objectives of this study were to investigate the assemblage and preferences of EPT genera on single and two species leaf packs and to test the hypothesis that leaf packs with many leaf species encourage higher EPT colonisation due to variation in nutrient contents hence resulting in faster decomposition.

## MATERIALS AND METHODS

### Sampling Site

The study was carried out at the Tupah River at N5°45.008′ E100°26.526′, located in a protected forest catchment of Gunung Jerai Forest Reserve in Kuala Muda district, state of Kedah, northern peninsular Malaysia. Image of the map can be found elsewhere (Suhaila *et al.* 2012). The river is 5.6 km long, with a mean depth of 0.32±0.05 meter and mean width of 4.14±0.28 meter. It enters the Merbok River that flows into the Straits of Malacca on the west coast of peninsular Malaysia. The Tupah River is fast flowing (0.56±0.16 m/s) with water pH ranging from 5.0 to 6.7 and an annual mean water temperature ranging from 22.8°C to 25.7°C. The river flows through lowland dipterocarp forests (100–200 meters above sea level). Tree species, such as *Shorea leprosula*, *Shorea ovata*, *Dipterocarpus* sp., *Dillenia* sp., *Pometia pinnata, Pongamia pinnata, Dolichandrone spathacea* and *Sindora sp.* are dominant in the*.* The river substrates are predominantly cobbles and gravels (~55%), with boulders (~45%).

### Litter Breakdown

Leaves from two tree species, *Pometia pinnata* (Family: Sapindaceae) and *Dolichandrone spathacea* (Family: Bignoniaceae) were selected for this study based on their abundance in the study area. The tree species were identified using keys of Corner (1988) and Ng and Sivasothi (1999) and verified by herbarium collection at the School of Biological Sciences, Universiti Sains Malaysia in Penang. The pinnate leaf of *P. pinnata* is 4–10 cm wide, thick, tough, glossy and relatively long (12–30 cm) while *D. spathacea* has pinnate thin, soft, glossy with a terminal leaflet and 2–4 pairs of opposite leaflets. Roughly similar sizes of mature green leaves were hand-picked from single trees to ensure homogeneity of leaves. They were air dried for three weeks. Two types of leaf packs were prepared; single species leaf pack using *P. pinnata* and two species leaf pack using a mixture of equal weight (1:1) of *P. pinnata* and *D. spathacea*. Approximately 8.0±0.2 g of leaves were weighed and placed into each cage. A single species leaf cage contained 8.0±0.2 g of *P. pinnata* or *D. spathacea* while a two species leaf cage was filled with 50% (4 g) *P. pinnata* and 50% (4 g) *D. spathacea*. All leaves were packed in wire cages (10 mm mesh) each measuring 15 cm × 10 cm × 5 cm as described by Mathuriau and Chauvet (2002).

To exclude the macroinvertebrates from the leaf pack, the cages were wrapped individually with a 1.5 mm mesh wire screen. These wrapped cages were the control leaf packs. The experimental cages were unwrapped and the EPTs have free access into and out of the cages. All cages (experimental and control leaf packs) were placed randomly in the river. Each cage was fastened to a meter long metal pole erected on the river bank to prevent it from drifting away during heavy rain. The leaves were assumed to decompose completely when the entire leaf fragments were broken to smaller than 5 mm in length (Mackay & Kersey 1985).

Fifty four experimental (allow entry to invertebrates) and 54 control (protected from invertebrates) leaf pack cages were completely submerged at random in the river. Each group of cages had equal numbers (18) of both single and two-species leaf packs. Three replicates from each treatment were retrieved weekly.

### Analysis of C and N in the Leaves

For assessing variation in nutrient content of the leaf species that might influence EPT preferences, the carbon and nitrogen contents of each leaf species were analysed. Fresh leaves of *P. pinnata* and *D. spataceae* were oven dried at 105°C for 24 h, weighed and ground to <0.5 mm in a Ball Mill (CMT, TI-100, Tokyo, Japan). By using a CN elemental analyser (EA, Thermo Finnigan NA 1500, North Clelmsford, MA, USA), two milligrams of each of the ground litter were analysed. The mean contents of both C and N were generated from three replicates and were estimated following the procedure of Shieh *et al.* (2007).

### Data Collection

Three cages of each single leaf and two leaf species in both experimental and control leaf packs were collected weekly. During retrieving, each cage was placed into a plastic bag containing a small amount of river water. Each leaf pieces in individual cage were removed, rinsed and insects in experimental cages were sorted and preserved in an 75% ethyl alcohol for subsequent documentation and recorded. Species keys of Malaysian aquatic insects were not available at the moment hence all EPTs were identified to respective genera using keys provided by Kenneth and Bill (1993), Morse *et al*. (1994), Wiggins (1996), Dudgeon (1999) and Yule and Yong (2004).

### Data Analysis

Richness metrics such as EPT taxa richness was used to analyse the EPT composition in all control and experimental leaf packs. Diversity Index (Shannon-Wiener, Simpson, Evenness) from all leaf packs was calculated using Species Diversity and Richness IV (SDR) version 4.1.2^®^ to describe and compare the EPT assemblages in different cages. The differences in abundance and diversity of EPT in weight loss among leaf packs types were compared by the non-parametric test (Kruskal-Wallis test) due to all data were not normally distributed (Kolmogorov-Smirnov test, *P* < 0.05) using the SPSS 20.0^®^.

The percentage remaining of leaf material lost over a period (%R) of time can be calculated by:

$$\%R=W(t_{f})/W(t_{i})\times 100$$

Where *W*(*t_i_*) is the initial weight of leaf material and *W*(*t_f_*) is the amount of material remaining after time t. This was assumed to follow a linear loss model proposed by Petersen and Cummins (1974) and can be expressed as % loss/day for comparison of species. For exponential models, the rate coefficient of litter breakdown (k) was estimated by the equation

$$-\text{k}={\text{log}}_{\text{e}}\;(\%R/100)/\text{t.}$$

k was possible because the leaf species had the initial and final weights measured. Leaf processing rate was categorised according to Petersen and Cummins (1974) based on the values of coefficient of litter breakdown rate (k) as ‘fast’ (>0.01 gm.day^−1^), ‘medium’ (0.005–0.010 gm.day^−1^) and ‘slow’ (<0.005 gm.day^−1^).

## RESULTS

### Litter Breakdown

**Figure 1:**
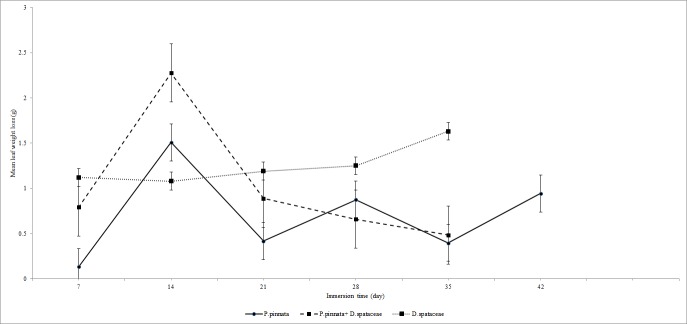
Leaf weight loss (mean ± SE) of control leaf packs in Tupah River, Kedah.

In control treatment (without EPT), the decomposition rate coefficient (k) in the two species leaf pack (k) was 0.047 g.day^−1^ and all leaves completely decomposed within 35 days (Fig. 1). In *P. pinnata* leaf pack the decomposition rate coefficient was slower (k=0.013 g.day^−1^) and decomposition took 42 days to complete while in *D.spataceae* leaf pack the decomposition rate coefficient was fastest (k = 0.21). These decomposition rates fell into fast decomposition category and fitted the exponential model with R^2^ = 0.37 for two species leaf pack, R^2^ = 0.17 for *P. pinnata* leaf pack and R^2^=0.86 for *D. spataceae* leaf pack (all at *P* = 0.05).

**Figure 2:**
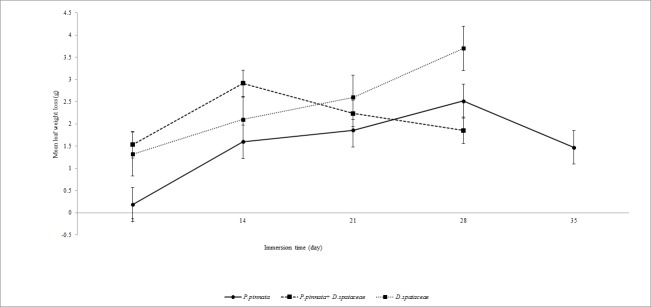
Leaf weight loss (mean ± SE) of experimental leaf packs in Tupah River, Kedah.

In the presence of EPT in experimental leaf packs (Fig. 2), faster decomposition of leaves was observed. Fifty percent of the weight in the single species leaf packs disappeared within 25 days but in two-species leaf packs, it only took half of the duration (12 days). The weight loss for two species leaf pack was faster than in single species leaf (χ^2^ = 11.771, *P* = 0.001). Leaf weight loss fitted the exponential model with R^2^=0.42 for two species leaf and R^2^ = 0.03 for *P.pinnata* leaf packs and R^2^ = 0.96 for *D. spataceae* leaf pack (all at *P* = 0.05).

In experimental leaf packs, average breakdown rate coefficient for two species leaf packs was 0.53 g.day^−1^, 0.025 g.day^−1^ for *P.pinnata* leaf packs and *D.spataceae* leaf packs was 0.46 g.day^−1^. After 28 days, the leaves in two species leaf packs were completely decomposed while all leaves in the single species leaf packs took 35 days to disintegrate. In both control and experimental leaf packs, decomposition of two species leaf pack was seven days faster than in both single species leaf pack and the leaves of *P.pinnata* decomposed slightly slower than *D.spathacea* leaves. In the presence of EPT in experimental leaf packs, leaves in both single species and two species leaf packs decomposed seven days faster than those in control (without EPT) leaf packs.

### Composition of C and N in the Leaf

*P. pinnata* had higher C:N ratio compared to *D. spathacea* (Table 1). *Dolichandrone spathacea* had double the amount of N thus higher quality leaf than *P. pinnata.*

### Communities of Ephemeroptera, Plecoptera and Trichoptera in Leaf Packs

During the period of study, all experimental cages were colonised mainly by EPTs and very few other insects (only chironomids and megalopterans) were occasionally caught. Therefore their presence in all cages were considered negligible and assumed not influencing the decomposition of the leaves. Twenty-three genera of EPT were found colonising leaf packs with 409 individuals in *P. pinnata* leaf packs, 166 individuals in *D.spathacea* and 385 individuals in two species leaf packs (Table 2). The EPT taxa richness in the two species leaf packs were higher (22) compared to both single species leaf packs (19 and 10). Both diversity indices and evenness (Pielou index) were slightly higher in *P.pinnata* leaf packs and two species leaf packs compared to *D.spathacea* leaf packs while the richness index (Menhinick index) was almost similar for all types of leaf packs.

Ephemeropterans preferred *D. spathacea* leaf packs (83.7%) followed by *P.pinnata* leaf packs (51%) and two species leaf packs (29%). Plecopterans were equally abundant in both types of leaf packs (21% in *P. pinnata* and 19% in two species leaf packs) but very low in *D. spathacea* leaf packs (2.4%) while more trichopterans colonised two species leaf packs (52.8%) (Table 3).

**Figure 3:**
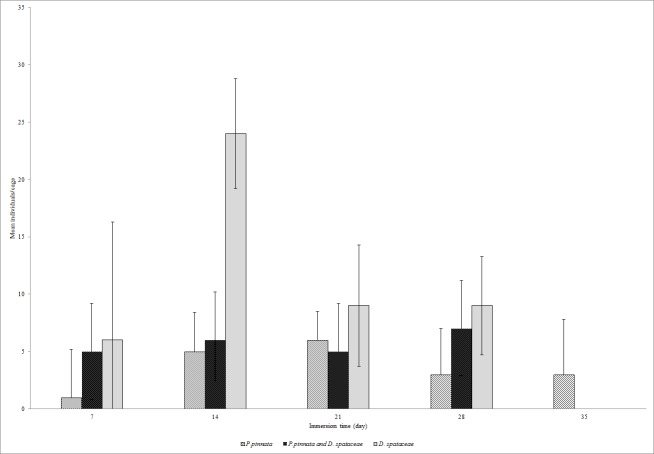
Abundance of Ephemeroptera, Plecoptera and Trichoptera (individual/cage) in experimental leaf packs during their breakdown in Tupah River, Kedah.

**Figure 4:**
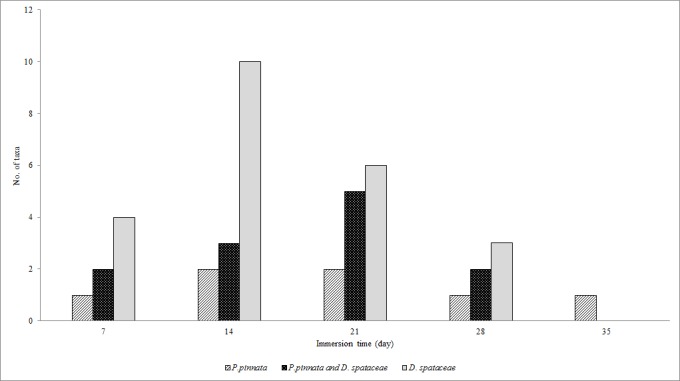
Total taxa of Ephemeroptera, Plecoptera and Trichoptera in experimental leaf packs during their breakdown in Tupah River, Kedah.

The abundance of EPT was very low at the beginning of the study. During the seven days, the *P.pinnata* leaf packs had only one individual per cage while in two species leaf packs, five individuals were collected however, 7 individuals were found in *D.spathacea* leaf packs (Fig. 3). The highest number of EPT was recorded on day 14 (24 individuals per cage) in *D.spathacea* leaf packs and on day 28 in two species leaf packs (7 individuals). There was no significant difference between EPT abundance and time of leaf immersion in the water (χ^2^ = 0.092, *P* = 0.762). In two species leaf pack, two genera were found during the first seven days then increased to five genera in the next 14 days (Fig. 4). From day 21 onward, only two EPT genera were found in the cage. In *P. pinnata* leaf pack, two genera were recorded on day 14 to 21 but only one genus was found thereafter.

## DISCUSSION

### Litter Breakdown

In this study, litter breakdown in control leaf packs represented natural decomposition in the absence of insects. Decomposition rate in *D. spataceae* leaf pack was found to be faster than in the single species (*P. pinnata*) leaf pack. Thicker tissue layer of *P. pinnata* leaves could contribute to slower decomposition of the leaves compared to that of *D. spataceae.* According to Quinn *et al*. (2000), tough leaves reduced litter breakdown rate because their surface layers had little abrasive loss (Irons *et al*. 1988; Stout, 1989). He found that tougher *Pinus radiata* leaves decomposed slower (k = 0.0036 g.day^−1^) than the softer black walnut leaves (k = 0.0390). In Tupah River, softer *D. spataceae* in the two species leaf pack could have rapidly broken down and less amount of *P. pinnata* (4 g compared to 8 g in single species leaf pack) shortened the overall litter breakdown process in this leaf pack.

In addition to leaf toughness, Ostrofsky (1997) reported that the levels of residual defensive compounds such as carbon amongst leaf species have contributed to variance in leaf processing rates. We observed in this study that *D.spataceae* with a C:N ratio of 14.3:1 decomposed faster than *Pinus pinnata* leaves with C:N ratio of 29.1:1. Mellilo *et al*. (1982) also reported that decomposition was fast in leaves with high N content because the saprotropic organisms (microbes) rapidly switched nitrogen into energy-rich rhizodeposits (Lekkerkerk *et al*. 1990). Suberkropp *et al*. (1976), Pearson *et al*. (1989), Stout (1989) and Mathuriau and Chauvet (2002) also reported comparable relationships between decomposition and nitrogen content. Softer leaves with high N content were more preferred by decomposer macroinvertebrates thus hastened the breakdown rates such as observed in the two species leaf packs of *P. pinnata* and *D. spataceae*. Greenwood *et al*. (2007) had reported a similar pattern when *Rhododendron maximum* leaves (C:N = 5.2) supported fewer macroinvertebrate (mean abundance = 9.9±5.5) compared to *Acer rubrum* leaves that contained high N (C:N = 3.2). More than 75 macroinvertebrates were found within the leaves. High C:N (lower quality) leaves were less preferred by the EPT because of low nutrient content or poor leaf quality.

According to Wantzen *et al*. (2008), the age of leaves influences their decomposition rates. Young leaves quickly decomposed because their cellulose structure is less stable. Using freshly picked mature leaves in this study ensures uniform maturity and homogeneity of all leaves because leaves do not undergo natural abscission at predetermined age in the tropics. Moreover, the timing of leaf loss in tropical riparian forests is highly variable (Dudgeon 2008).

Previous studies have shown that breakdown of leaf litter is more rapid in low-order tropical rivers compared to similar order rivers in temperate areas (Dudgeon 1982; Benstead 1996) although tropical leaves generally are more recalcitrant and have higher levels of secondary compounds than leaves from temperate deciduous trees (Li & Dudgeon 2009). Leaves in tropical rivers naturally decompose faster than in temperate areas because of higher water temperatures (Suberkropp *et al*. 1976; Dudgeon & Wu 1999; Mathuriau & Chauvet 2002; Abelho *et al*. 2005) that favour microbial activities (Irons *et al*. 1994; Mathuriau & Chauvet 2002; Gonçalves *et al*. 2006). It was reported that increasing water temperature in rivers from 25 to 35°C would increase bacterial respiration from 26 to 63% (Sand-Jensen *et al*. 2007).

### Colonization of EPT in Two Types of Leaf Packs

EPT abundance was higher in the tougher and coarser surface *P. pinnata* but the slower decomposition of single species leaf packs compared to two species leaf packs was presumably related to the poor quality of *P. pinnata* leaves. Lin *et al*. (2002) reported that low nutrient quality leaves decompose slowly and the nutrients are not available to invertebrates for a longer period before it has been conditioned (Shieh *et al*. 2007).

Decomposition rates were faster in in the presence of EPT, emphasising their role in leaf breakdown in headwater streams. The decomposition rate of *P.pinnata* (k = 0.025 g.day^−1^) was faster in the presence of EPT than in protected leaf packs (k = 0.013 g.day^−1^). The same occurred in the two species treatment (k = 0.53 g.day^−1^ vs. k = 0.047 gm.day^−1^). Naturally, the leaves are rapidly colonised by EPTs for food sources as well as for their habitats (Abelho & Graça 1998; González & Graça 2003; Stewart *et al*. 2003; Wan Mohd Hafezul *et al*. 2016).

In this study, Ephemeroptera was the dominant and diverse order among the EPTs recorded during the entire decomposition process. Most of them were collector-gatherers that mainly fed on FPOM from allocthonous resources. They benefited from the FPOM produced by the decomposition process thus their numbers were always high in the leaf packs. Ephemeroptera was more diverse in *P. pinnata* leaf pack compared to two species leaf pack. During the first seven days after immersion, 80% of total Ephemeroptera colonised the *P. pinnata* and *D. spataceae* leaf and 65% colonised the two species leaf packs. Higher abundance of Ephemeroptera have been similarly recorded previously in other tropical rivers (Mathuriau & Chauvet 2002; Moulton & Magalhães 2003; Gonçalves *et al*. 2006; Suhaila *et al.* 2012; Al-Shami *et al*. 2013). Number of shredders was very low in this study and none of them are from Ephemeroptera order. Those found shredders were from Plecoptera (2 genera) and Trichoptera (3 genera).

Meanwhile, Plecoptera diversity and abundance was low compared to Trichoptera and Ephemeroptera in both leaf packs. The plecopterans were mainly predators and very few were shredders (*Indonemoura* and *Cryptoperla*). Their numbers increased after seven days of leaf immersion in the two species leaf packs, which probably correlated with their shredding activity immediately after the microbes softened the leaves. Predator stoneflies probably appeared in the leaf packs when more preys were available there. Generally, Plecoptera are less abundant and less diverse in most of the tropical streams as documented earlier by Rosemond *et al.* (1998), Yule and Yong (2004), and Yule *et al*. (2009).

Trichoptera was diverse in two species leaf pack and their abundance increased after seven days of leaf immersion in the water. Among them, the calamoceratids and leptocerids (shredders) preferred softer leaves that have been partially digested by microbial enzymes and fungal activities (Quinn *et al*. 2000) coinciding with its higher abundance and diversity on those days. Many trichopterans were collector-gatherers, feeding on FPOM generated by the decomposition process and they were found in the cages until the leaves were completely decomposed.

Several recent studies on leaf litter processing in tropical streams (Mathuriau & Chauvet 2002; Parnrong *et al*. 2002; Cheshire *et al*. 2005; Gonçalves *et al*. 2006; Wantzen & Wagner 2006; Shieh *et al*. 2007) have provided valuable insight regarding this process in the tropical rivers. However more field studies concerning leaf species preferences by aquatic insects especially among the EPT taxa are desirable. The interaction of river retentiveness and leaf decay rates can guide the riparian management to recommend more tree plantings to increase river supply of terrestrial organic carbon because in-stream nutrient dynamics are influenced by the litter inputs from riparian vegetation (Quinn *et al*. 2000; Che Salmah *et al*. 2013).

## CONCLUSION

The leaves completely decomposed within 35 days in two species leaf pack and 42 days in single species leaf pack in the control packs. Insects of the order EPT helped in decomposing the leaf but not from the outset. Early stage of EPT colonisation on leaves mostly intricate the ephemeropterans. With the interference of EPT, the leaf breakdown rate was faster in both leaf packs, with the two species leaf pack completely decomposed within 28 days and both the single species in 35 days, showing that EPT had played a significant role in litter breakdown. Soften leaves attracted more EPT because soft leaves did not require more time to be softened by microbe and the leaves are available to the shredders. Mixture of many leaf species does encourage EPT assemblages as different type of species will have different coefficient rate. Many stages of litter breakdown in one leaf or in one microhabitat can be used as habitat and also as food source.

## Figures and Tables

**Table 1:** Carbon (C) and nitrogen (N) (% of dry weight ± SE) contents and the C:N ratio of *P. pinnata* and *Dolichandrone spathacea* leaves.

Constituent	*Pometia pinnata*	*Dolichandrone spathacea*
C	44.67 ± 0.30	44.79 ± 0.40
N	1.54 ± 0.05	3.13 ± 0.04
C:N	29.1:1	14.3: 1

**Table 2:** Ephemeroptera, Plecoptera and Trichoptera assemblages in the experimental leaf packs.

Metric	*P. pinnata*	*P. pinnata* + *D. spataceae*	*D. spataceae*
Abundance	409	385	166
EPT taxa richness	19	22	10
Shannon-Wiener Index (H′)	2.40	2.38	1.18
Menhinick Index (R^2^)	0.60	0.59	0.78
Pielou Evenness Index (*J*′)	0.963	0.958	0.486

**Table 3:** Relative abundance of Ephemeroptera, Plecoptera, Trichoptera colonising experimental leaf packs in Tupah River.

Order	Family	Genus	*P.pinnata* (%)	*P.pinnata* + *D.spataceae* (%)	*D.spataceae* (%)
Ephemeroptera	Heptageniidae	*Thalerosphyrus*	3.9	1.3	0
	Baetidae	*Baetis*	26.7	13.2	8.6
	Baetidae	*Platybaetis*	1.2	0.5	1.0
	Tricorythidae	*Tricorythus*	3.9	2.1	0
	Caenidae	*Caenis*	8.8	3.6	71.7
	Heptageniidae	*Campsoneuria*	3.9	4.7	0
	Leptophlebiidae	*Habrophlebiodes*	2.2	2.9	1.8
		*Choroterpes*	0	0	0.6
	Ephemerellidae	*Crinitella*	0	0.3	0
Plecoptera	Nemouridae	*Indonemoura*	0.2	0	0
	Perlidae	*Kamimuria*	1.2	0.3	0
	Perlidae	*Neoperla*	18.8	17.9	0
	Perlidae	*Phanoperla*	1.2	0.3	2.4
	Peltoperlidae	*Cryptoperla*	0	0.3	0
Trichoptera	Ecnomidae	*Ecnomus*	1.5	0.3	0
	Hydropsychidae	*Cheumatopsyche*	13	27	0
	Hydropsychidae	*Hydropsyche*	10.5	18.4	0
	Hydropsychidae	*Macrostemum*	0.2	0.5	0
		*Diplectrona*	0	0	0.6
	Calamoceratidae	*Ganonema*	1.5	0.5	0
	Rhyacophilidae	*Rhyacophila*	0.2	0.5	1.8
	Philopotamidae	*Chimarra*	0	2.9	0
	Lepidostomatidae	*Lepidostoma*	0	1.8	0
	Leptoceridae	*Setodes*	0.7	0.3	0
		*Oecetis*	0	0	1.2
	Odontoceridae	*Marilia*	0.2	0.6	0
	Seriscostomatidae	*Gumaga*	0	0	4.8
	Molannidae	*Molanodes*	0	0	5.4

**Table 4:** The leaf pack rate coefficients (k) and % loss/day for selected leaf species in control and experimental cages.

Leaf species	k	% loss/day
*P.pinnata* (control)	0.013	0.06
*P.pinnata* + *D.spataceae* (control)	0.047	0.24
*D.spataceae* (control)	0.210	3.4
*P.pinnata* (experimental)	0.025	0.05
*P.pinnata* + *D.spataceae* (experimental)	0.053	0.75
*D.spataceae* (experimental)	0.146	9.5
